# Supplementary material for: Structural basis of QueC-family protein function in qatABCD anti-phage defense
Source: bioRxiv. 2025 Sep 3:2025.09.03.674047. Preprint. [Version 1] doi: 10.1101/2025.09.03.674047 (PMC12424860; doi:10.1101/2025.09.03.674047)
Supplement: 3 [file NIHPP2025.09.03.674047v1-supplement-3.pdf]

bioRxiv preprint doi: https://doi.org/10.1101/2025.09.03.674047; this version posted September 3, 2025. The copyright holder for this preprint (which was not certified by peer review) is the author/funder, who has granted bioRxiv a license to display the preprint in perpetuity. It is made available under aCC-BY 4.0 International license.

Supplementary Figures

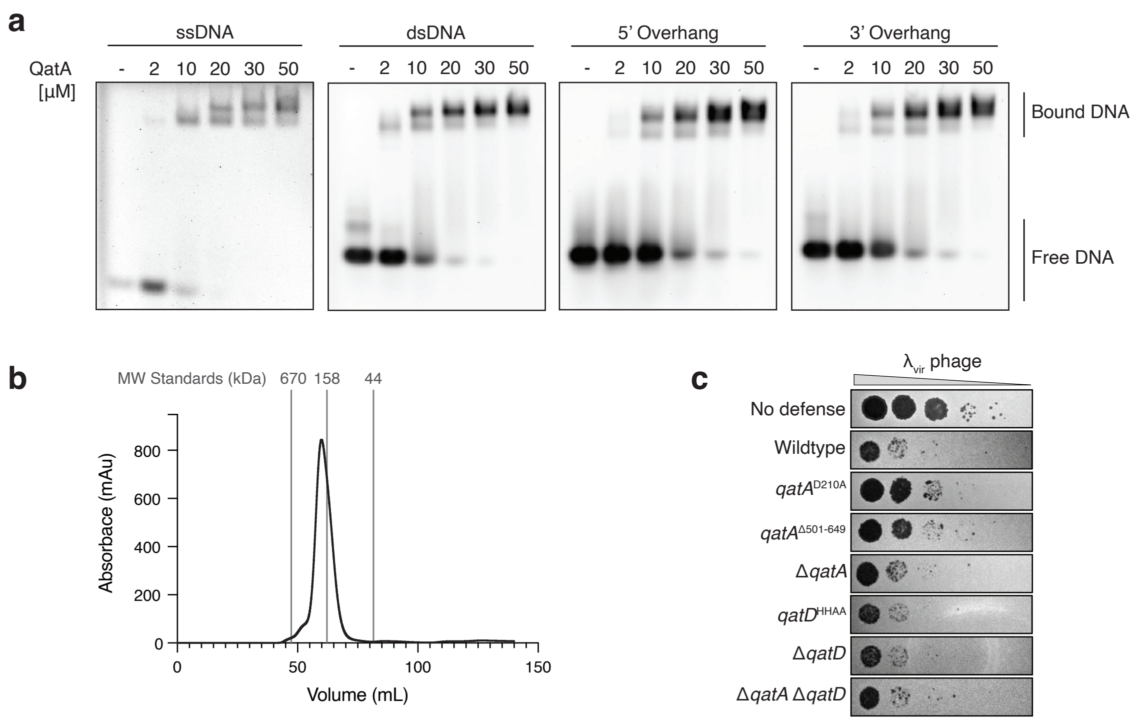

Supplementary Figure 1 | QatA and QatD in qatABCD-mediated phage defense

a, Electrophoretic mobility shift assays of QatA on ssDNA, dsDNA, 5′ overhang, and 3′ overhang substrates run on 2% TB-agarose gels stained with ethidium bromide. Protein concentrations are expressed as µM of QatA monomer. **b,** Size exclusion chromatography trace of His-tagged QatA expressed in the full qatABCD operon. Calculated molecular weight of QatA monomer is 72 kDa and QatA dimer is 144 kDa. Peak elution volumes of molecular weight standards are indicated by vertical grey lines. Thyroglobulin, 670 kDa; γ-globulin, 158 kDa; ovalbumin, 44 kDa. **c,** Representative plaque assays of *E. coli* expressing GFP control (no defense) or *P. aeruginosa* qatABCD with the indicated (n=4). QatD H15A/H17A (qatD^{HHAA}).

bioRxiv preprint doi: https://doi.org/10.1101/2025.09.03.674047; this version posted September 3, 2025. The copyright holder for this preprint (which was not certified by peer review) is the author/funder, who has granted bioRxiv a license to display the preprint in perpetuity. It is made available under aCC-BY 4.0 International license.

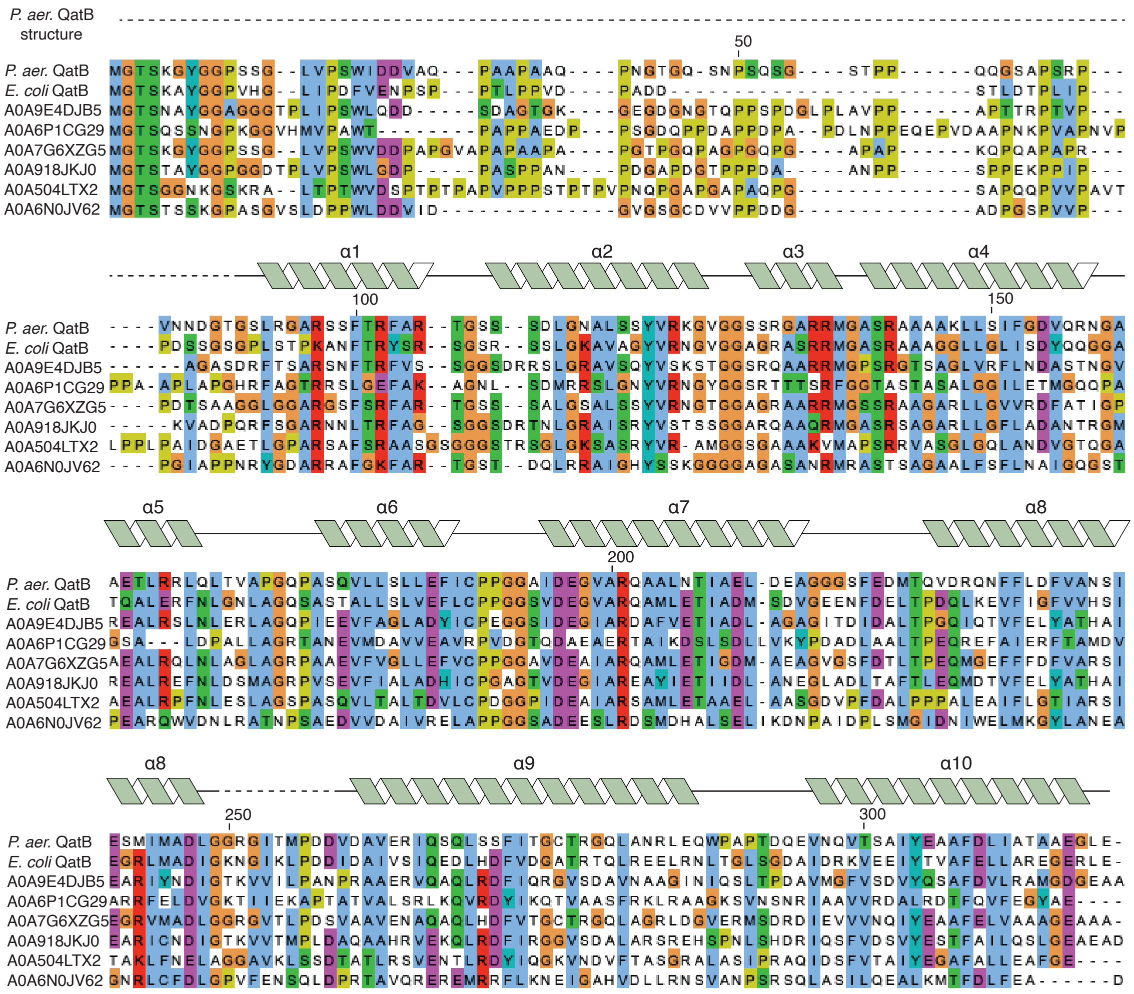

Supplementary Figure 2 | QatB alignment

Sequence alignment of representative QatB sequences (UniProtKB accession numbers shown). Secondary structure is annotated from *P. aeruginosa* QatB structure in complex with QatC. Alignment is colored using ClustalX color scheme based on side chain properties.

bioRxiv preprint doi: https://doi.org/10.1101/2025.09.03.674047; this version posted September 3, 2025. The copyright holder for this preprint (which was not certified by peer review) is the author/funder, who has granted bioRxiv a license to display the preprint in perpetuity. It is made available under aCC-BY 4.0 International license.

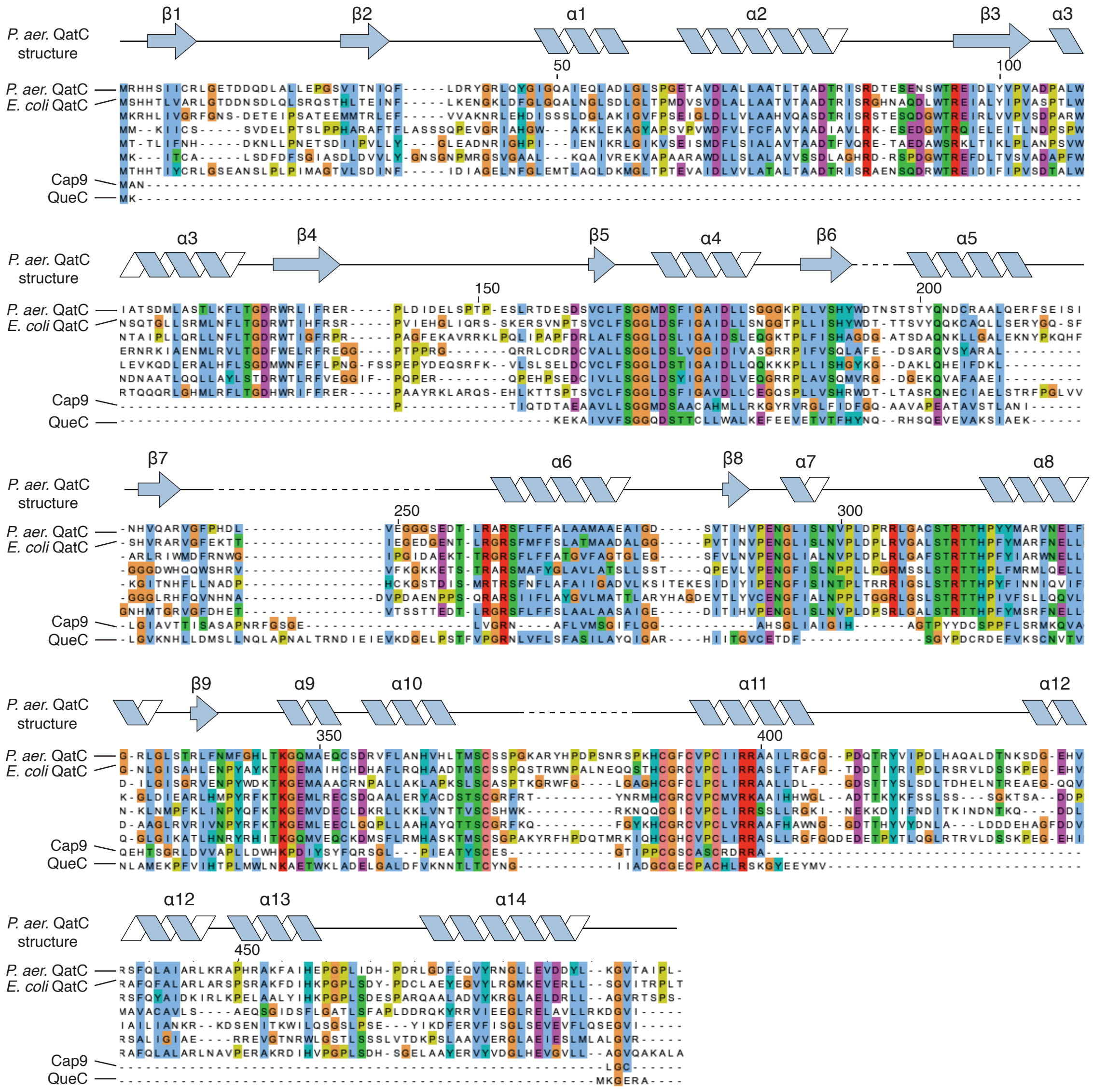

Supplementary Figure 3 | QatC alignment

Sequence alignment of representative QatC sequences (UniProtKB accession numbers: A0A2P6WBR3, A0A1E2V6V1, A0AA91FJV8, A0A158EPR1, A0A7X2LQZ7), *Rhizobiales sp.* Cap9, and *B. subtilis* QueC. Secondary structure is annotated from *P. aeruginosa* QatC structure in complex with QatB. Alignment is colored using ClustalX color scheme based on side chain properties.
